# Sequencing and analysis of the complete mitogenome of *Doriprismatica atromarginata* (Cuvier, 1804)

**DOI:** 10.1080/23802359.2019.1660276

**Published:** 2019-09-06

**Authors:** Thinh Dinh Do, Mustafa Zafer Karagozlu, Van Quan Nguyen, Chang-Bae Kim

**Affiliations:** a Department of Biotechnology, Sangmyung University, Seoul, Korea;; b Institute of Marine Environment and Resources, Vietnam Academy of Science and Technology, Haiphong, Vietnam

**Keywords:** Mitogenome, Chromodorididae, *Doriprismatica atromarginata*, phylogenetic relationships

## Abstract

*Doriprismatica atromarginata* is a common nudibranch in the tropical and sub-tropical Indo-Pacific region. In this study, the complete mitogenome of *D. atromarginata* from Vietnam was recorded for the first time. The circular mitogenome had a size of 14,421 bp and consisted of 37 genes (13 protein-coding genes, two ribosomal RNA genes, and 22 tRNA genes). Phylogenetic tree based on amino acid sequences of coding genes demonstrated that *D. atromarginata* has sister group relationship with the genus *Chromodoris*.

Chromodorididae is a large family of the order Nudibranchia with over 300 recorded species (Johnson and Gosliner 2012). Based on a combination of morphological and molecular analyses, several cryptic and pseudo-cryptic species in this family have been detected recently (Matsuda and Gosliner 2018). Mitogenome is well known as a common marker for the resolution of taxonomic controversies (Gissi et al. 2008; Duchêne et al. 2011). However, to date, there are only seven mitogenomes of two genera (*Chromodoris* and *Hyselodoris*) from the family Chromodorididae recorded. In this study, we sequenced and analysed the mitogenome from a Chromodorididae species, *Doriprismatica atromarginata*.

*D. atromarginata* collection was conducted by Scuba diving in Hon Me island (19°22′3.50″N, 105°54′59.44″E), Vietnam in July 2018. Upon collection, the specimen was vouchered and stored in the Department of Biotechnology, Sangmyung University (Voucher no. SMU0034). The methods for DNA extraction and mitogenome generation were described previously (Do et al. 2019). For the investigation of phylogenetic relationships, a tree was constructed based on amino acid sequences of protein-coding genes (PCGs). The neighbour-joining method with 1000 bootstrap replicates in MEGAX software was used for tree construction (Kumar et al. 2018).

The *D. atromarginata* mitogenome (GenBank accession number: MN171300) was 14,421 bp in size, containing 13 PCGs, two ribosomal RNA genes, and 22 tRNA genes. Overall nucleotide composition was 29.4% A, 39.9% T, 17.5% G, and 13.2% C. Similar to recorded mitogenomes of Chromodorididae, *D. atromarginata* mitogenome is composed of 24 genes encoded on H-strand and 13 genes encoded on L-strand. Of 13 PCGs, *nd5* was the longest gene (1680 bp) while *atp8* (156 bp) was the shortest gene. For ribosomal RNA, 12S rRNA gene was 751 bp in length, and 16S rRNA gene was 1175 bp in length. There were 13 overlapping regions which ranging from 3 to 33 bp. The longest overlapping region was located between tRNA^Val^ and 16S rRNA.

For start codon, only *nd6* started with ATT and *nd4l* started with ATA, the remaining genes started with ATG. For stop codon, two genes (*nd5* and *cytb*) were terminated with TAG while remaining genes were terminated with TAA.

The phylogenetic tree showed relationships between nudibranchs in the family Chromodorididae (Figure 1). It indicated that *D. atromarginata* have a close relationship with *Chromodoris* species (*C. orientalis*, *C. annae*, *C. magnifica,* and *C. quadricolor*).Itpositioned as sister to the genus *Chromodoris* and they formed a clade with the genus *Hypselodoris*. Our result is in agreement with the pattern in previous report by Johnson and Gosliner (2012) based on mitochondrial markers (16S rRNA and *cox1*). This is the first mitogenome record for the species and the third genus for the family Chromodorididae. For a better understanding of the phylogeny of the family, more mitogenomes from different genera should be sequenced and analysed.

**Figure 1.**
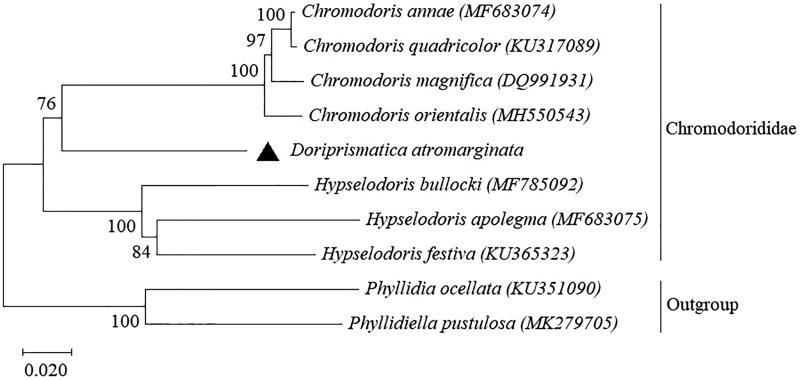
The phylogenetic tree of *D. atromarginata* in the Chromodorididae family based on amino acid sequences of mitochondrial protein-coding genes. GenBank’s sequences with accession numbers were obtained for the analysis. Triangular symbol represents *D. atromarginata* mitogenome. *Phyllidia ocellate* and *Phyllidiella pustulosa* (*Phyllidiidae*) were used for the outgroup.
